# Telemedicine in Pediatrics: Systematic Review of Randomized Controlled Trials

**DOI:** 10.2196/22696

**Published:** 2021-02-24

**Authors:** Aashaka C Shah, Sherif M Badawy

**Affiliations:** 1 University of Illinois College of Medicine Chicago, IL United States; 2 Division of Hematology, Oncology, Neuro-Oncology and Stem Cell Transplant Ann & Robert H Lurie Children's Hospital of Chicago Chicago, IL United States; 3 Department of Pediatrics Northwestern University Feinberg School of Medicine Chicago, IL United States

**Keywords:** telemedicine, telehealth, pediatrics, COVID-19, coronavirus, pandemic, digital, eHealth, mHealth, mobile health

## Abstract

**Background:**

Telemedicine modalities, such as videoconferencing, are used by health care providers to remotely deliver health care to patients. Telemedicine use in pediatrics has increased in recent years. This has resulted in improved health care access, optimized disease management, progress in the monitoring of health conditions, and fewer exposures to patients with illnesses during pandemics (eg, the COVID-19 pandemic).

**Objective:**

We aimed to systematically evaluate the most recent evidence on the feasibility and accessibility of telemedicine services, patients’ and care providers’ satisfaction with these services, and treatment outcomes related to telemedicine service use among pediatric populations with different health conditions.

**Methods:**

Studies were obtained from the PubMed database on May 10, 2020. We followed the PRISMA (Preferred Reporting Items for Systematic Reviews and Meta-Analyses) guidelines. In this review, we included randomized controlled trials from the last 10 years that used a telemedicine approach as a study intervention or assessed telemedicine as a subspecialty of pediatric care. Titles and abstracts were independently screened based on the eligibility criteria. Afterward, full texts were retrieved and independently screened based on the eligibility criteria. A standardized form was used to extract the following data: publication title, first author’s name, publication year, participants’ characteristics, study design, the technology-based approach that was used, intervention characteristics, study goals, and study findings.

**Results:**

In total, 11 articles met the inclusion criteria and were included in this review. All studies were categorized as randomized controlled trials (8/11, 73%) or cluster randomized trials (3/11, 27%). The number of participants in each study ranged from 22 to 400. The health conditions that were assessed included obesity (3/11, 27%), asthma (2/11, 18%), mental health conditions (1/11, 9%), otitis media (1/11, 9%), skin conditions (1/11, 9%), type 1 diabetes (1/11, 9%), attention deficit hyperactivity disorder (1/11, 9%), and cystic fibrosis–related pancreatic insufficiency (1/11). The telemedicine approaches that were used included patient and doctor videoconferencing visits (5/11, 45%), smartphone-based interventions (3/11, 27%), telephone counseling (2/11, 18%), and telemedicine-based screening visits (1/11, 9%). The telemedicine interventions in all included studies resulted in outcomes that were comparable to or better than the outcomes of control groups. These outcomes were related to symptom management, quality of life, satisfaction, medication adherence, visit completion rates, and disease progression.

**Conclusions:**

Although more research is needed, the evidence from this review suggests that telemedicine services for the general public and pediatric care are comparable to or better than in-person services. Patients, health care professionals, and caregivers may benefit from using both telemedicine services and traditional, in-person health care services. To maximize the potential of telemedicine, future research should focus on improving patients’ access to care, increasing the cost-effectiveness of telemedicine services, and eliminating barriers to telemedicine use.

## Introduction

Telemedicine is a broad term that describes the use of technology in health services for patients and families [1-3]. Such services include teleeducation, telecounseling, and telecommunication platforms that enhance the effectiveness and reach of health care [1,2]. Physicians and other health care providers mainly use telemedicine technology to conduct remote patient visits [1]. This is especially true in the field of pediatrics, given that patients and families frequently face obstacles such as a limited number of pediatric specialists and barriers to long-distance travel [4-7]. Recent advances in pediatric telemedicine have made it possible to deliver pediatric services to medically underserved regions and low-income countries [2,8,9]. Overall, this has led to improved access to health care and the fast assessment, monitoring, and treatment of patients [2,10]. Numerous studies have reported that these benefits, along with the cost-effectiveness of videoconferencing visits (ie, compared to that of in-person visits), have improved the quality of life of patients and their caregivers [8-12]. However, even with new telemedicine technology, barriers to telemedicine access still exist, including the need for strong internet connections, software, and equipment [3,8,10]. Furthermore, studies have shown that the maintenance of telemedicine software is costly, especially in rural areas where such software can be especially useful [8]. The professional and ethical challenges that come with internet-based health care affect patients and physicians [3,13]. Patients and their caregivers can be hesitant to partake in telemedicine encounters due to their desire to see a physician in person, the need for insurance reimbursement, or their attitudes toward technology [1].

Due to the many benefits that telemedicine encounters can provide to patients and physicians, telemedicine services have been used more frequently in recent years [1]. The COVID-19 pandemic has highlighted several important benefits, challenges, and barriers in health care delivery [5,14-18]. Stay-at-home orders, reductions in the number of elective procedures, the loss of jobs, and people’s avoidance of hospitals and emergency rooms have made it increasingly difficult for patients to maintain their health care needs during the pandemic [14,17,19,20]. Telemedicine technologies can be especially beneficial during the pandemic, as they can be used to minimize people’s exposure to patients with illnesses and provide an on-demand alternative to traditional, in-person visits [15,17,21-23]. Although children who test positive for COVID-19 typically exhibit mild symptoms, routine health services are still an important aspect of a child’s well-being [24]. Patients with chronic conditions or those who exhibit risk factors for severe disease (eg, asthma or allergies) can be evaluated via telemedicine modalities for ensuring proper disease management [24].

The future uses of telemedicine technology may include remote patient monitoring, triage, and the implementation of telemedicine services in rural settings or low-income countries [1,8,10]. The goals of telemedicine research include reducing the cost of telemedicine services and optimizing the use of telemedicine technology across different settings [2,8]. These goals are achievable, especially with the growing amount of evidence that supports the feasibility, acceptability, and efficacy of many digital interventions (eg, telehealth approaches) [25-29].

The unique challenges resulting from the COVID-19 pandemic, limited accessibility of pediatric health care in rural areas, management of childhood chronic illnesses, lack of pediatric specialists (ie, compared to the number adult care specialists), and difficulties in traveling with children have highlighted the usefulness and importance of telemedicine modalities for the pediatric population [4-7]. Recent studies and reviews have suggested that telemedicine is a cost-effective, feasible, and beneficial mode of delivering health care for a variety of medical conditions, such as diabetes, heart disease, and depressive disorder [30-33]. Telemedicine’s beneficial role in neonatal intensive care unit patient monitoring and pediatric obesity management have also been noted in reviews [10,34]. This review aims to compare the use of telemedicine modalities to that of standard care modalities and determine whether telemedicine procedures can replace standard, face-to-face care procedures. Specifically, the objective of this review is to systematically evaluate the most recent evidence on the feasibility and accessibility of telemedicine services, patients’ and care providers’ satisfaction with these services, and treatment outcomes related to the use of telemedicine among pediatric populations with different health conditions.

## Methods

### Study Design

We followed the PRISMA (Preferred Reporting Items for Systematic Reviews and Meta-Analyses) guidelines to report on evidence from the studies that were included in this systematic review [35-37]. The PRISMA checklist is shown in Multimedia Appendix 1. We conducted a literature search on the PubMed database on May 10, 2020. The following four keywords were used to conduct the PubMed database search: “telemedicine pediatrics,” “telehealth pediatrics,” “telemedicine kids,” and “telehealth kids.” These search terms accounted for related Medical Subject Headings terms, which allowed us to capture a broad range of relevant articles from the database. The “randomized control trial” and “last ten years” filters were applied to all four searches, which were based on each keyword. All articles from the literature search were collected, and duplicate articles were excluded from this review. Titles and abstracts were independently screened based on the eligibility criteria. Articles that did not meet the inclusion criteria were excluded from this review. Afterward, full texts were retrieved and independently screened based on the eligibility criteria. Disagreements were settled by discussion.

### Eligibility Criteria

Original randomized controlled trials that were published after 2010 and used telemedicine modalities for different pediatric populations were eligible for this review. No restrictions were placed on the language, condition, setting, or country of a trial. The inclusion criteria included original research papers, randomized controlled trials, pediatric populations (ie, general pediatric care or a subspecialty of pediatric care), and a focus on telemedicine as a study intervention. This review was limited to randomized controlled trials so that we could assess studies with the highest quality of evidence. In order to focus on recent telemedicine advances and the current uses of telemedicine technology, eligible studies were limited to those that were published within the last 10 years.

### Data Extraction and Synthesis

A standardized form was used for data extraction. The data items in this form included the following: publication title, first author’s name, publication year, participants’ characteristics, study design, the technology-based approach that was used, intervention characteristics, study goals, and main study findings. Synthesized data were qualitatively analyzed. ACS conducted the data extraction and SMB conducted a review of the final data.

### Quality and Strength of Evidence

The quality of evidence from the studies that were analyzed in this review was independently evaluated by using the Grading of Recommendations, Assessment, Development, and Evaluation (GRADE) approach [38]. This approach involves assigning an initial quality level rating to a study based on the study design. Randomized controlled trials were all assigned an initial quality level rating of high. The quality level of a study can then be upgraded or downgraded based on the various factors listed in the GRADE guidelines. Factors for downgrading a study’s quality level included limitations in the study design and the execution of a study, indirect evidence, inconsistent results, imprecise results, and bias. Quality levels could be upgraded if a study had large effect sizes or dose gradients. Disagreements on GRADE quality levels were settled by discussion.

## Results

### Literature Search

**Figure 1:**
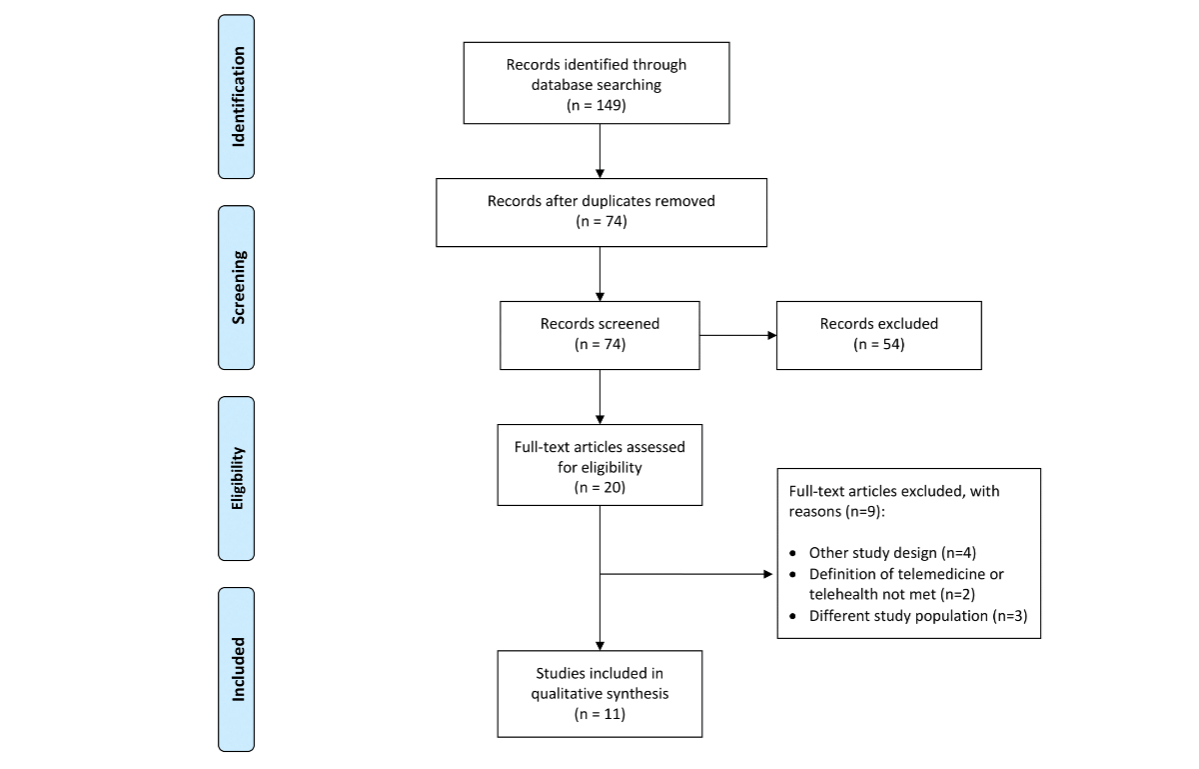
Flow diagram of the study inclusion and exclusion process.

We conducted a literature search on the PubMed database on May 2020, and this initial literature search yielded a total of 149 references. The “randomized control trial” and “past ten years” filters were applied to all four searches. After excluding duplicates, 74 references remained. The titles and abstracts of all 74 articles were screened, and of these 74 articles, 20 met all the predefined inclusion criteria. Full texts were retrieved from these 20 articles. Afterward, 9 articles were excluded. A total of 11 articles were included in this review [39-49]. The reasons for excluding full-text articles are stated in the PRISMA study flowchart (Figure 1).

### Study Characteristics

The characteristics of all included studies are reported in Tables 1 and 2. The studies in this review involved a broad range of health conditions, including asthma (2/11, 18%) [46,47], obesity (3/11, 27%) [40,41,44], mental health conditions (1/11, 9%) [48], otitis media (1/11, 9%) [49], skin conditions (1/11, 9%) [43], type 1 diabetes (1/11, 9%) [42], attention deficit hyperactivity disorder (ADHD) (1/11, 9%) [45], and cystic fibrosis–related pancreatic insufficiency (1/11, 9%) [39]. Of the 11 included studies, 9 (82%) were conducted in the United States of America [39-41,43-48], 1 (9%) was conducted in Italy [42], and 1 (9%) was conducted in Finland [49]. All studies were published in English. Studies’ sample sizes ranged from 22 participants [44] to 400 participants [46]. Of the 11 included studies, 4 (36%) had a small sample size (ie, <50 participants) [41,43,44,49], and another 4 (36%) had a sample size of >200 participants [45-48]. The average or median age of participants ranged from 21 months [49] to 17.7 years [42]. Of the 11 studies, 1 (9%) reported that the median age of participants was <3 years [49], and 2 (18%) reported that the average age of participants was >13 years [41,42]. Most trials (7/11, 64%) had a greater proportion of male participants than female participants [39,42,45-49]. All study designs were classified as either randomized controlled trials (8/11, 73%) [39,41-46,49] or cluster randomized controlled trials (3/11, 27%) [40,47,48], as per the inclusion criteria of this review. Follow-up periods ranged from 60 days [49] to 5 years [46]. Of the 11 included studies, 8 (73%) had a follow-up period that ranged between 6 months and 12 months [40-42,44-48], and 1 (9%) did not conduct a participant follow-up [43]. Based on the GRADE criteria, the quality of evidence from most studies was low (4/11, 36%) [41,43,47,48] or moderate (6/11, 55%) [40,42,44-46,49]. Of the 11 studies, only 1 (9%) had a quality rating of high [39]. The telemedicine techniques that were used in the studies included patient and doctor telemedicine visits (5/11, 45%) [40,41,45-47], telemedicine-based screening visits (1/11, 9%) [48], smartphone-based interventions (3/11, 27%) [42,43,49], and telephone counseling (2/11, 18%) [39,44]. Detailed descriptions of the telemedicine techniques that were used in the included studies are discussed in the “Telemedicine Approaches” section. The primary and secondary outcome measures of each study are included in Table 2. Most primary outcomes focused on changes in patients’ symptoms (8/11, 72%) [35,39-42,44-47], the time effectiveness of telemedicine (1/11, 9%) [48], or the concordance between in-person and telemedicine diagnoses (2/11, 18%) [43,49].

**Table 1:** Characteristics of participants in all included studies.

Source (year, country)	Number of participants	Mean age of participants	Female participants, %
Cocker et al (2019, United States) [48]	Total: 342 Control: 178 Intervention: 164	8.6 years	38.3
Erkkola-Anttinen et al (2019, Finland) [49]	Total: 41 Immediate group: 20 Delayed group: 21	21 months^a^	42
Perry et al (2018, United States) [47]	Total: 363 Control group: 183 Intervention group: 180	9.6 years^a^	44
Halterman et al (2018, United States) [46]	Total: 400 Control group: 200 Intervention group: 200	7.8 years	38.25
O’Connor et al (2017, United States) [43]	Total: 40 Control group: 20 Intervention group: 20	6.96 years	55
Di Bartolo et al (2017, Italy) [42]	Total: 182 Control group: 90 Intervention group: 92	17.7 years	48.9
Fleischman et al (2016, United States) [41]	Beginning of study: Total: 40 Control group: 21 Intervention group: 19 End of study: Total: 33 Control group: 19 Intervention group: 14	14.3 years	77.5
Rhodes et al (2017, United States) [44]	Total: 22 Low GLb group: 11 Low-fat group: 11	Low GL group: 8.1 years Low-fat group: 8.2 years	Low GL group: 54.5 Low-fat group: 63.6
Stoep et al (2017, United States) [45]	Total: 223 Control group: 112 Intervention group: 111	9.23 years	29.9
Davis et al (2016, United States) [40]	Total: 103 Control group: 61 Intervention group: 42	9.14 years	55.34
Powers et al (2015, United States) [39]	Total: 78 Control group: 42 Intervention group: 36	3.8 years	43

^a^Median used instead of mean.

^b^GL: glycemic load.

**Table 2:** Summary of study characteristics and the quality of evidence from all included studies.

Source (year, country)	Health condition	Study design	Telemedicine approach	Outcome measures	Follow-up period	Quality of evidence^a^
Cocker et al (2019, United States) [48]	Mental health	Cluster RCT^b^	Video orientations and videoconferencing screening visits with a mental health clinic	Primary: completion of screening visit Secondary: time from referral to screening visit and completion of intake visit	6 months	Low
Erkkola-Anttinen et al (2019, Finland) [49]	Otitis media	RCT	At-home otoscopy videos via smartphone	Primary: exclusion of otitis media Secondary: diagnostic quality of videos and effects of teaching interventions	60 days	Moderate
Perry et al (2018, United States) [47]	Asthma	Cluster RCT	Asthma education and monitoring via a telemedicine approach	Primary: number of symptom-free days Secondary: peak flow meter use, medication adherence, quality of life, self-efficacy, lung function, and asthma knowledge	6 months	Low
Halterman et al (2018, United States) [46]	Asthma	RCT	School-based telemedicine visits	Primary: number of symptom-free days Secondary: number of days with symptoms, use of rescue medication, and number of days with limited activity	7-9 months for intervention and up to 5 years after enrollment	Moderate
O’Connor et al (2017, United States) [43]	Skin condition	RCT	Parents used a smartphone to photograph their child’s skin condition for direct patient-to-physician telemedicine.	Primary: Concordance between in-person and photograph-based diagnoses Secondary: parents’ willingness, image quality, and effect of photograph instructions	None	Low
Di Bartolo et al (2017, Italy) [42]	Type 1 diabetes	RCT	Glucose meters were able to sync with a phone app, which can directly send information to health care workers. Patients were able to contact physicians via email, SMS text messaging, or telephone.	Primary: changes in hemoglobin A_1c_ levels Secondary: number of patients who self-monitored their blood glucose levels and patients’ quality of life	12 months	Moderate
Fleischman et al (2016, United States) [41]	Obesity	RCT	Televisits with obesity specialists and teleconsults between physicians and specialists	Primary: changes in BMI Secondary: waist circumference, triceps skinfold, blood pressure, dietary glycemic load, and physical activity	12 months	Low
Rhodes et al (2017, United States) [44]	Obesity	RCT	Dietary counseling via telephone	Primary: changes in glycemic load and total number of calories in fat Secondary: total energy intake	12 months	Moderate
Stoep et al (2017, United States) [45]	Attention deficit hyperactivity disorder	RCT	Telepsychiatry sessions via video counseling	Primary: changes in distress, as measured by a variety of questionnaires Secondary: patient health, caregiver strain, parenting stress, and family empowerment	25 weeks	Moderate
Davis et al (2016, United States) [40]	Obesity	Cluster RCT	Physicians delivered behavioral group interventions to families via a telemedicine approach.	Primary: BMI *z* score Secondary: feasibility measures, parents’ BMIs, 24-hour dietary recall, behavioral checklist scores, feeding assessment scale scores, and accelerometer data	8 months	Moderate
Powers et al (2015, United States) [39]	Cystic fibrosis and pancreatic insufficiency	RCT	Parts of both treatments were delivered via telephone.	Primary: changes in energy intake Secondary: changes in weight *z* scores and changes in height *z* scores	18 months	High

^a^Quality ratings are based on the Grading of Recommendations, Assessment, Development and Evaluation criteria.

^b^RCT: randomized controlled trial.

### Telemedicine Approaches

Telemedicine approaches widely varied across all included studies. Several studies (5/11, 45%) involved traditional patient and doctor visits [40,41,45-47]. These studies conducted videoconferencing visits instead of in-person physician visits [40,41,45-47]. Of the 11 studies, 3 (27%) used telemedicine interventions that involved the use of a smartphone [42,43,49], and 2 (18%) required parents to perform a task with their smartphone prior to the doctor visit [43,49]. One of these tasks required a parent to perform an at-home smartphone otoscopy of a patient’s ear [49], and another required a parent to take a picture of a patient’s skin condition in the clinic waiting room [43]. Another smartphone telemedicine approach involved using a new blood glucose meter, which synced data from patients’ phones with an app that was able to notify their physicians [42]. Furthermore, two studies used telephone counseling as their principal telemedicine approach [39,44]. In the first study, telephone dietary consultations were made available to participants [44]. The second study involved telephone nutrition counseling and telephone-based education on child behavior management for parents [39]. Additionally, one study used videoconferencing and telemedicine methods in the intervention group and telephone communication methods in the control group [40]. Another study conducted a screening visit via a telemedicine approach [48]. In this study, a mental health clinic conducted an initial screening visit via videoconferencing instead of a traditional, in-person visit [48]. Detailed descriptions of telemedicine approaches are included in Textbox 1.

**Summary of the telemedicine approaches that were used in all included studies.**

**Cocker et al (2019) [48]**

- This was a study on mental health.
- A community mental health clinic conducted an initial screening visit via videoconferencing instead of via telephone.
- After receiving a mental health referral from the primary care physician, parents watched an introduction video about the community mental health clinic.
- Parents returned to the health center and connected with the community mental health clinic coordinator via videoconferencing to determine their eligibility for a screening visit.

**Erkkola-Anttinen et al (2019) [49]**

- This was a study on otitis media.
- Patients were randomized into either the immediate and delayed teaching groups.
- The immediate teaching group received instructions on how to use a smartphone otoscope before the study began.
- The delayed teaching group received instructions after the first week of the study.
- Parents performed a bilateral smartphone otoscopy on their child for a minimum of 5 days during the first week.
- After the first week, bilateral otoscopy was performed (1) once per week if the child was not experiencing symptoms; (2) every day if child was experiencing respiratory symptoms; (3) every day for 1 week following a diagnosis of acute otitis media; (4) any day the child was experiencing ear pain; and (5) on days of physician visits.
- Bilateral otoscopy videos were sent to the study physician via iMessage, email, or WhatsApp.

**Perry et al (2018) [47]**

- This was a study on asthma.
- Students participated in five age-appropriate asthma education telemedicine sessions with an allergist, respiratory therapist, or asthma educator.
- These sessions involved the use of a standard, prewritten script.
- Parents or caregivers participated in two telemedicine asthma education sessions that were conducted at a school.
- Nurses participated in two telemedicine asthma education sessions that were conducted at a school.
- If 3 or more sessions were missed, education was delivered via telephone, and education materials were mailed ahead of time.
- Patients were assessed via telemonitoring on months 0 and 3, and asthma medication information was provided by parents on months 3 and 6.
- Caregiver-reported outcomes were measured via telephone interviews on months 0, 3, and 6.

**Halterman et al (2018) [46]**

- This was a study on asthma.
- Initial asthma assessments for patient and caregivers were conducted via a telemedicine approach.
- A telemedicine assistant entered baseline patient data into the electronic health record system, and a clinician completed the visit within 3 days (ie, from the office or via real-time videoconferencing).
- Afterward, the clinician contacted patients’ caregivers by phone or videoconference to discuss initial patient symptoms, treatment plans, and asthma education.
- If a patient’s primary care physician did not conduct telemedicine visits, another physician was assigned as the patients’ primary physician during the study. Information was forwarded to the original primary care physician.
- Follow-up assessments were conducted via a telemedicine approach every 4-6 weeks.
- All telemedicine visits were reviewed by a nurse to ensure that proper guidelines were followed.

**O’Connor et al (2017) [43]**

- This was a study on skin conditions.
- Parents took photographs of their child’s skin condition with their smartphone in the examination room.
- In this study, 50% of parents received photography instructions and the other 50% did not.
- Photographs were uploaded to electronic medical records.

**Di Bartolo et al (2017) [42]**

- This was a study on type 1 diabetes.
- Patients who were allocated to the IBGStar (Sanofi US) group received training on how to use the IBGStar machine.
- These patients were able to measure their blood glucose levels with the IBGStar machine at home and sync the readings to an app on their smartphone.
- Data on the app could be directly shared with health care providers.
- All participants in this study were able to contact their physician via email, SMS text messaging, or telephone.

**Fleischman et al (2016) [41]**

- This was a study on obesity.
- All participants attended in-person visits with their primary care physician every 3 months.
- All participants’ primary care physicians conducted a teleconsultation with an obesity specialist 1 week before the visit to discuss obesity treatment.
- Group 1 attended obesity specialist televisits and primary care physician visits for the first 6 months of the study. In the following 6 months, participants only visited their primary care physician in person.
- Group 2 only visited their primary care physician in person for the first 6 months of the study. In the following 6 months, primary care physician visits were supplemented with obesity specialist televisits.

**Rhodes et al (2017) [44]**

- This was a study on obesity.
- All participants received weekly dietician telephone consultations for 5 consecutive weeks.
- Consultation sessions were recorded, and several sessions were screened to ensure that they adhered to the study protocol.
- This study had a standardized procedure for addressing any missed consultations.

**Stoep et al (2017) [45]**

- This was a study on attention deficit hyperactivity disorder.
- Families in the telemedicine group underwent a total of 6 combined telemedicine and in-person treatment sessions.
- Videoconferencing was used to deliver child psychiatry treatment and therapy.
- Therapists provided parents with education on attention hyperactivity disorder at the end of each telepsychiatry session.
- All of the sessions were recorded, and a subset of sessions was reviewed to ensure that they were accurate and guideline compliant.
- Therapists were provided with asynchronous telehealth training modules on how to most effectively deliver attention deficit hyper activity education to caregivers.
- These telehealth modules involved viewing recordings of interventions on an asynchronous website.
- Recordings were obtained from volunteer families.
- The control group received 1 telepsychiatry session at the beginning of the study.
- The telepsychiatrist recommended treatment to patients’ primary care physicians based on this visit.
- Primary care physicians recommend this treatment, along with any other treatment that they felt would be beneficial, to their patients.

**Davis et al (2016) [40]**

- This was a study on obesity.
- The schools in this study were randomly allocated into either the telephone or telemedicine groups.
- Telephone and telemedicine sessions were held at schools and focused on family-based cognitive behavioral therapy.
- The telephone group sat around a speakerphone, which was used to connect with the research team during the sessions.
- Speakerphones were provided if the school did not already have one.
- The telemedicine group used the audio and video functions of a television screen to communicate with the research team.

**Powers et al (2015) [39]**

- This was a study on cystic fibrosis and pancreatic insufficiency.
- The behavioral and nutritional treatment group received individualized nutritional counseling and parent education on child behavioral management.
- Treatment/education sessions and data collection were conducted via an in-person approach or a telehealth approach (ie, telephone).
- If a family did not consistently report on their child’s dietary data, a nurse would contact the family via telephone in order to retrieve data.
- The education and attention control group were given educational resources that were related to cystic fibrosis and pancreatic insufficiency. Individualized counseling was not provided to this group. In-person visits and telehealth (ie, telephone) techniques were used to conduct appointments and collect data.

### Study Outcomes

#### Summary of Study Outcomes

Descriptions of study outcomes are reported in Textbox 2. Additional details on these study outcomes are included in Multimedia Appendix 2 [39-49].

**Summary of the main findings and outcomes of all included studies.**

**Cocker et al (2019) [48]**

- This was a study on mental health.
- The initial screening visit was completed by a greater proportion of patients in the telemedicine group (132/164, 80.49%) than in the control group (114/178, 64.04%).
- Patients in the telemedicine referral group required more days to complete the initial screening visit (mean 23.6 days) than patients in the control group (mean 17.1 days).
- No significant difference was observed in the proportion of patients who completed the recommended intake visit after the screening visit between the two groups (telemedicine group: 93/116, 80.2%; control group: 81/97, 83.5%; *P*=.51).
- Based on the adjusted analysis, no significant difference was observed in the time from referral to the screening visit between the two groups (*P*=.62).
- Compared to parents in the control group, those in the telemedicine group reported higher satisfaction with the referral system and the care that they received.
- No significant differences were observed in patients’ quality of life (ie, after 6 months) between both groups (*P*=.82).

**Erkkola-Anttinen et al (2019) [49]**

- This was a study on otitis media.
- A video or image was obtained during 98% (1472/1500) of all parent-performed examinations (median video length=18 seconds).
- In total, 67% (867/1293) of all videos were of sufficient diagnostic quality.
- Diagnoses could be made for 56% (486/867) of videos that were of sufficient diagnostic quality.
- Diagnoses could only be made for 8% (35/426) of the videos that were of insufficient diagnostic quality.
- Diagnoses could be made for 40% (521/1293) of all videos.
- Acute otitis media diagnoses could be confirmed or excluded for 87% (609/699) of all videos that were obtained during respiratory infection.
- In total, diagnoses could be confirmed or excluded with 99% (495/501) of the videos that were of sufficient diagnostic quality.
- In total, diagnoses could be confirmed or excluded with 58% (114/198) of the videos that were of insufficient diagnostic quality.
- During week 1 of the intervention, the immediate teaching group was taught how to perform otoscopy and the delayed teaching group was not. There were significantly more videos that were of sufficient diagnostic quality in the immediate teaching group (95/152, 62%) than in the delayed teaching group (39/179, 22%) (*P*<.001).
- One week after the delayed teaching group received their education session, 64% (85/133) of their videos were of sufficient diagnostic quality.
- In total, 24% (10/41) of families believed that smartphone otoscopy was a burden.
- In total, 83% (34/41) of families considered conducting smartphone otoscopies on a daily basis.

**Perry et al (2018) [47]**

- This was a study on asthma.
- No significant difference was observed in the number posttreatment symptom-free days between the intervention and usual care groups (*P*=.51).
- Patients in both groups still had uncontrolled asthma at the end of treatment.
- Compared to the intervention group, the usual care group had significantly higher scores in the family activity domain of the Child Health Survey for Asthma (*P*=.02).
- Compared to the usual care group, the intervention group had a significantly greater percentage of patients that used a peak flow meter (*P*<.001).
- Compared to the usual care group, the intervention group had a significantly greater percentage of patients who were compliant with posttreatment asthma medication (*P*=.03).
- There was no significant difference in the baseline quality-of-life scores between both treatment groups (*P*=.06).

**Halterman et al (2018) [46]**

- This was a study on asthma.
- Children in the telemedicine group had significantly more postintervention symptom-free days (mean 11.6 days) than children in the control group (mean 10.97 days) (*P*=.01).
- The intervention group had fewer symptom days, symptom nights, and limited activity days than the control group.
- Compared to the control group, the telemedicine group had a greater proportion of patients who were prescribed preventive medication (control group: 132/196, 67%; telemedicine group: 181/199, 91%).
- In the final follow-up longitudinal visit, the telemedicine group had 0.85 more symptoms than the control group, and a significant correlation was observed between treatment efficacy and time (*P*<.02).
- Decreases in exhaled nitric oxide levels were greater in the telemedicine group than in the control group (mean difference=−5.54).
- Caregivers’ quality of life improved in both groups; there was no significant difference in caregivers’ quality of life between both groups (95% CI −0.08 to 0.37).
- In total, 95.7% (361/377) of patients reported that the program was helpful, and 96.5% (365/367) reported that they would partake in another similar program.

**O’Connor et al (2017) [43]**

- This was a study on skin conditions.
- The median photograph quality rating score was 9.
- The concordance between photograph diagnosis and in-person diagnosis for all photographs was 83% (33/40).
- The mean quality rating score for photographs with a diagnosis was 8.9, whereas the mean quality rating score for photographs with no diagnosis was 7.0.
- The group that received photography instructions had a higher average image quality score and a higher mean number of images than the group that did not receive instructions, but this was not statistically significant.
- No significant difference was observed in the concordance of diagnosis between the group that received photograph instructions and the group that did not receive instructions (*P*=.68).
- Parents’ willingness to use teledermatology services was measured on a scale of 1 (ie, not willing) to 10 (ie, very willing). The median response score was 8.

**Di Bartolo et al (2017) [42]**

- This was a study on type 1 diabetes.
- The telemedicine and control groups exhibited reduced hemoglobin A_1c_ levels after treatment; there was no significant difference between the two groups (*P*=.051).
- Patients who self-monitored their blood glucose levels exhibited reduced hemoglobin A_1c_ levels at 6 months posttreatment.
- Patients who did not self-monitor their blood glucose levels only exhibited minor changes in hemoglobin A_1c_ levels at 6 months posttreatment.
- Patients in the telemedicine group exhibited greater decreases in hemoglobin A_1c_ levels at 6 months posttreatment than the control group (*P*=.25).
- The control group started using the experimental telemedicine meter at 6 months posttreatment. At 12 months posttreatment, the control group exhibited decreases in hemoglobin A_1c_ levels (*P*=.24).
- At 12 months posttreatment, the experimental group’s hemoglobin A_1c_ levels remained stable (ie, compared to their hemoglobin A_1c_ levels at 6 months posttreatment).
- There were no significant differences in quality-of-life measures between both groups at 6 months and 12 months posttreatment (*P*=.23).

**Fleischman et al (2016) [41]**

- This was a study on obesity.
- Group 1 (ie, patients who attended primary care physician visits and specialist televisits) exhibited greater decreases in BMI *z* scores after 3 months than Group 2 (ie, patients who only attended primary care physician visits) (*P*=.049).
- The BMIs in group 1 significantly decreased after 6 months (*P*<.001), while the BMIs in Group 2 did not (*P*=.08). No significant differences were observed in BMIs between the two groups (*P*=.23).
- After 6 months, group 1 only attended primary care physician visits and Group 2 attended primary care physician visits and specialist televisits.
- The baseline BMIs in group 1 were significantly different from those after 9 months (*P*.004) and 12 months (*P*=.03).
- The baseline BMIs in group 2 were significantly lower than those after 12 months (*P*=.03).
- If given the opportunity to choose between obesity specialist televisits or in-person visits, 14 patients would choose televisits and 7 had no preference.

**Rhodes et al (2017) [44]**

- This was a study on obesity.
- There were no significant differences in dietary fat content (ie, before and after treatment) between or within the two groups (*P*=.68).
- After treatment, the low glycemic load group had lower glycemic loads than the low-fat group (*P*=.003).
- There were no significant differences in posttreatment glycemic loads between both groups (*P*=.06).
- The low glycemic load group exhibited a significant decrease in total energy intake levels after treatment (*P*<.005).
- The low glycemic load group had significantly lower posttreatment total energy intake levels than the low-fat group (*P*=.001).
- There were no significant differences in changes in total energy intake levels (ie, from baseline to after treatment) between both groups (*P*=.06).

**Stoep et al (2017) [45]**

- This was a study on attention deficit hyperactivity disorder.
- Caregivers in both the Children’s Attention Deficit Hyperactivity Disorder Telemental Health Treatment Study (CATTS) and augmented primary care groups showed improvements in caregiver distress by the end of the study.
- Caregivers in the CATTS group had significantly lower Parenting Stress Index (*P*<.01; Cohen *d*=0.59), Patient Health Questionaire-9 (*P*<.05; Cohen *d*=0.27), and Cognitive Skills Quotient (*P*<.001; Cohen *d*=0.45) scores after 25 weeks of treatment compared to those at baseline.
- Caregivers in the CATTS group also had significantly higher Falls Efficacy Scale scores after 25 weeks of treatment (*P*<.01; Cohen *d*=−0.44).

**Davis et al (2016) [40]**

- This was a study on obesity.
- The satisfaction scores between the telemedicine and telephone groups were not considerably different.
- There were no significant differences in changes in patients’ BMIs (ie, pretreatment to posttreatment) between the telemedicine and telephone groups (*P*>.05).
- There were no significant differences in changes in parents’ BMIs (ie, pretreatment to posttreatment) between the telemedicine and telephone groups (*P*>.05).

**Powers et al (2015) [39]**

- This was a study on cystic fibrosis and pancreatic insufficiency.
- After treatment, the control group had significantly lower energy intake levels than the behavioral and nutritional treatment group (*P*<.001).
- After treatment, there were no significant differences in weight *z* scores between the two groups (*P*=.25).
- After treatment, the control group exhibited greater decreases in height *z* scores than the behavioral and nutritional treatment group (*P*=.49).
- During the follow-up, the behavioral and nutritional treatment group had greater average energy intake levels than the control group (*P*=.02).
- At follow-up, there were no significant differences in weight *z* scores between the two groups (*P*=.61).

#### Effects of Telemedicine on Asthma Symptoms

Perry et al [47] and Halterman et al [46] used a school-based telemedicine approach to aid patients with managing their asthma symptoms. Perry et al [47] reported that there were no significant differences in the number of symptom-free days (SFDs) between the telemedicine and usual care groups (*P*=.51), while Halterman et al [46] reported a significant increase in the number of SFDs in the telemedicine group compared to that in the control group (*P*=.01). Perry et al [47] reported that there was a significant increase in medication adherence (*P=*.03) and peak flow meter use (*P*<.001) in the telemedicine group compared to those in the usual care group. Furthermore, Halterman et al [46] reported that the telemedicine group had a greater proportion of patients who were prescribed preventative medicine (181/199, 91%) compared to the control group (132/196, 67%). The telemedicine group also had lower hospitalization rates (14/199, 7%) than the control group (29/196, 15%). Additionally, patients in the telemedicine group had a significantly higher number of SFDs in the follow-up longitudinal visit than the control group (*P*<.02) [46]. Both Perry et al [47] and Halterman et al [46] reported no significant differences in quality-of-life scores between the groups at the end of their studies. In terms of satisfaction, most parents in the Halterman et al study [46] stated that they found the program helpful (361/377, 95.7%) and would partake in another similar program (365/377, 96.5%). Furthermore, parents in the telemedicine group were more likely to learn more about asthma medication (152/193, 78.8%) than parents in the control group (111/184, 60.3%) [46].

#### Effects of Telemedicine on Weight Management and Energy Intake

Fleischman et al [41], Rhodes et al [44], and Davis et al [40] investigated the role of telemedicine in weight management by conducting specialist televisits, telephone dietary counseling, and physician telemedicine interventions, respectively. In the Fleischman et al study [41], obesity specialists found that each group’s BMIs significantly decreased 6 months after the telemedicine phase of the study (group 1: *P=*.006; group 2: P=.03). Rhodes et al [44] showed that a low–glycemic index diet significantly decreased the posttreatment total energy intake levels of both groups (*P<*.005). Furthermore, the low glycemic load group exhibited greater decreases in total energy intake levels than the low-fat diet group (*P=*.001). However, there were no significant differences in changes in total energy levels (ie, from the beginning of treatment to the end of treatment) between the two groups (*P=*.06) [44]. Similarly, Davis et al [40] reported that there were no significant differences in changes in patients’ and parents’ BMIs (ie, from baseline to after treatment) within (P>.05) and between (*P>*.05) the two groups. In the Fleischman et al study [41], most patients (14/21, 67%) stated that they prefer televisits over in-person specialist visits, and patients in the telemedicine group found the program more helpful than patients in the control group (*P=*.06). Alternatively, Davis et al [40] did not observe a significant difference in satisfaction scores between the telemedicine and telephone groups [40]. Powers et al [39] tracked the effects that telehealth-based nutritional counseling and education had on patients with cystic fibrosis–related pancreatic insufficiency. Powers et al [39] reported that the control group had significantly lower posttreatment energy intake levels (*P<*.001) and greater decreases in height *z* scores (*P=*.49) than the treatment group. No significant differences were observed in posttreatment weight *z* scores between the two groups (*P*=.25) [39].

#### Effects of Telemedicine on Diabetes Management

Di Bartolo et al [42] measured changes in patients’ blood glucose levels by using a traditional blood glucose meter and the IBGStar blood glucose meter (Sanofi US). This study showed that both groups exhibited reductions in hemoglobin A_1c_ (HbA_1c_) levels. There were no significant differences in HbA_1c_ levels between the two groups at the end of treatment (*P=*.051) [42]. The number of patients who self-monitored their blood glucose levels was comparable between the two groups (*P=*.85) [42]. The self-monitoring of blood glucose levels was associated with decreases in HbA_1c_ levels [42]. The telemedicine group used the experimental IBGStar meter and reported greater decreases in HbA_1c_ levels at 6 months posttreatment than those who used the traditional meter (*P=*.25) [42]. Even at 12 months posttreatment, the experimental group’s HbA_1c_ levels were stable (ie, compared to their HbA_1c_ levels at 6 months posttreatment) [42]. There were no significant differences in quality-of-life measures between both groups at 6 and 12 months posttreatment [42]. Participants in the telemedicine group contacted their physician (ie, via SMS text messaging, telephone call, or email) more frequently than the control group [42].

#### Effects of Telemedicine on Screening Efficiency

Cocker et al [48] compared the efficiency of telemedicine mental health screening visits to that of in-person screening visits. Although screening visits were completed by a greater percentage of patients in the telemedicine group (132/164, 80%) than in the in-person group (114/178, 64%), patients in the telemedicine group required longer times to complete the screening visit (telemedicine group: mean 23.6 days; in-person group: mean 17.1 days) [48]. The mode of delivery for the screening visit did not have a considerable effect on the percentage of patients who completed the in-person intake visit [48]. Patients’ quality of life did not differ between the two groups, but patients in the telemedicine group reported higher satisfaction with the screening process than the in-person group [48].

#### Effects of Telemedicine on Patients’ and Caregivers’ Quality of Life

Stoep et al [45] assessed the effects of ADHD therapy and caregiver education (ie, both were provided via a telemedicine approach) on parents’ quality of life (ie, parents from the Children’s ADHD Telemental Health Treatment Study). After 25 weeks, parents in the telemedicine group exhibited significant decreases in their Parenting Stress Index (*P*<.01), Patient Health Questionaire-9 (*P*<.05), and Client Satisfaction Questionnaire (*P*<.001) scores, as well as significant increases in their Falls Efficacy Scale scores (*P*<.01) [45]. At the end of the study, parents experienced improvements in different domains of caregiver distress, including parenting stress (41%), caregiver depression (48%), caregiver strain (43%), and family empowerment (26%). These percentages refer to the effects of treatment on caregiver outcomes (ie, changes in children’s symptoms/roles) [45]. Reductions in the number of patient’s oppositional defiant disorder symptoms correlated with decreased levels of caregiver distress [45].

#### Effectiveness of Parent Telemedicine Education

Erkkola-Anttinen et al [49] and O’Connor et al [43] conducted studies that required parents to learn telemedicine techniques for documenting their child’s health condition. Erkkola-Anttinen et al [49] provided caregivers with education on performing a smartphone otoscopy of a patient’s ear. O’Connor et al [43] instructed parents to take a photograph of a patient’s skin condition. Erkkola-Anttinen et al [49] showed that acute otitis media diagnoses that were confirmed or excluded based on videos from parents who received smartphone otoscopy instructions (495/501, 99%) were more accurate than those based on videos from parents who did not receive instructions (114/198, 58%). In the Erkkola-Anttinen et al study [49], a considerable difference was observed in the quality of videos from the teaching and nonteaching groups. However, O’Connor et al [43] reported that there was no significant difference in the concordance of photograph-based and in-person diagnoses between parents who received instructions and parents who did not receive instructions (*P=*.68). The mean quality rating score of photographs from which a diagnosis could be made (8.9) was higher than that of photographs from which a diagnosis could not be made (7.0) [43]. Similarly, Erkkola-Anttinen et al [49] reported that a diagnosis could be made with 56% (486/867) of otoscopy videos that were of sufficient diagnostic quality. However, a diagnosis could only be made with 8% (35/426) of videos that were not of sufficient diagnostic quality. In the O’Connor et al study [43], parents’ willingness to use teledermatology services was measured on a scale of 1 (ie, not willing) to 10 (ie, very willing). The median rating was 8 [43].

## Discussion

### Principal Findings

The evidence from this review suggests that telemedicine visits for pediatric care may be comparable to and occasionally more beneficial than in-person visits. In this review, 11 studies that met all listed inclusion criteria were identified. All included studies were randomized controlled trials that assessed the use of telemedicine in pediatrics. The following eight health conditions were assessed: asthma, obesity, otitis media, mental health conditions, skin conditions, ADHD, type 1 diabetes, and cystic fibrosis–related pancreatic insufficiency. According to the GRADE criteria, the quality of evidence from almost all studies (10/11, 91%) was either low or moderate. Most low or moderate ratings were due to limitations in study design and implementation and the indirectness of evidence. The quality of evidence from one study was high. Most studies conducted videoconferencing visits instead of traditional, in-person physician visits. Other telemedicine interventions that were used included smartphone-based apps, telephone counseling, and web-based screening visits.

Overall, although the impact of telemedicine on pediatric health care was modest, telemedicine interventions showed promise. Studies on school-based telemedicine interventions for asthma had contradictory results for the effects of telemedicine on asthma SFDs [46,47]. However, parents were satisfied with these interventions and noticed improvements in outcome measures, such as asthma education, medication adherence, and the number of preventative medicine prescriptions [46,47]. Similarly, although studies about the impact of telemedicine on weight management had mixed results, patients reported that they preferred televisits over in-person visits or had no preferences for the two methods [39-41,44]. Patients also reported that they were more satisfied with telemedicine approaches than with mental health screening visits [48]. Furthermore, parents’ (ie, those of children with ADHD) quality of life improved after attending web-based therapy and education sessions [45]. This suggests that telemedicine services can be used to supplement in-person visits. Studies have also reported that parent education on telemedicine techniques for monitoring and documenting children with health conditions is a feasible approach that is acceptable to caregivers [43,49]. Additionally, patients who use telemedicine-based blood sugar monitoring devices have reported that they contact their physicians more frequently. This suggests that telemedicine technology can be used to supplement digital approaches for monitoring chronic health conditions [42].

Recently published literature has suggested that telemedicine approaches in general pediatric practice can be used to provide alternatives to traditional patient visits, increase people’s access to health care, and reduce the number of existing disparities [50-52]. One of the goals of recent research has been to improve the standards of telemedicine services so that they can provide higher quality care with lower costs [50,51]. The management of chronic health conditions is a realm of pediatrics in which telemedicine approaches have shown promise, especially when they are used in conjunction with in-person approaches [7,53].

Health care has been rapidly evolving to adapt to the ongoing COVID-19 pandemic, and telemedicine has become an important mechanism of health care delivery [19]. A study found that telemedicine visits in urgent care and nonurgent care facilities have increased by 135% and 4345%, respectively [19]. Many pediatric patient portals have also been updated and improved to include telehealth features [54]. The use of telemedicine during the COVID-19 pandemic not only protects patients and providers from unnecessary exposure to patients with illnesses, but also conserves personal protective equipment, which should be saved for essential encounters [55]. New telemedicine technologies, such as chatbots that provide conversation-like interactions, are being used to triage patients and screen for COVID-19 symptoms [56]. However, due to the increased use of telemedicine technology in hospitals and clinics, these technologies need to be evaluated so that people can understand their effects on patients, workers, health care systems, and insurance companies [57].

The timely management of pediatric chronic illnesses, such as obesity, allergies, and genetic diseases, is paramount to providing patients and their families with the best care, especially during the COVID-19 pandemic [24,58,59]. Web-based telemedicine visits have been used to help manage chronic conditions and related medications [24,58,59]. Additionally, during the COVID-19 pandemic, glucose monitoring software has been used to regularly record type I diabetes symptoms [60]. Common symptoms, such as migraines, can worsen during times of stress, and telemedicine can aid with providing care and limiting the need to visit a hospital [61].

Telemedicine is also being used in specific pediatric subspecialty settings. In surgery, telemedicine modalities have been used to preoperatively diagnose patients, perform surgery (ie, with robotic devices), or postoperatively monitor patients [62]. Pediatric gastroenterologists have also used telemedicine to supplement in-person visits and monitor chronic conditions (eg, inflammatory bowel disease) [63]. Furthermore, due to the limited number of pediatric subspecialty physicians in certain regions, telemedicine referrals are being used to optimize the accessibility of subspecialty resources [64]. A survey study that was conducted at a pediatric headache clinic in San Francisco, California reported that all included families found telemedicine visits to be more convenient than in-person visits. These families also stated that they would choose to use a telemedicine method again [65]. Families of children with many different health conditions have shown considerable interest in telemedicine visits, and most of these families possess sufficient technology for attending these visits [52].

Pediatric patients in rural communities face distinct challenges, such as limited access to subspecialty care and long commutes to clinics. However, these challenges can be overcome with telemedicine interventions [4,66-69]. Pediatricians from rural areas of the United States have advocated for telemedicine, as it can help with maintaining patient relationships and improving the accessibility of subspecialty care [70]. Telemedicine can provide a convenient platform that patients (eg, those from rural communities) can use to obtain the health care that they need, minimize travel time, and reduce waiting times for appointments [4,66-69].

The use of telemedicine in adult medical care is similar to that in pediatric care. Web-based patient monitoring via telemedicine modalities allows intensive care unit physicians to check the status of multiple patients at any time and place [71]. In one study, neurology patients were monitored with web-based electrocardiogram and electroencephalogram machines [72]. Telemedicine technologies can also be used to improve preprocedural instructions (eg, bowel preparation instructions for a colonoscopy) and reduce the time needed for providing adequate education [73].

### Strengths and Limitations

This systematic review has multiple strengths. First, we followed recommendations for rigorous systematic review methodologies [35-37]. Second, language and country filters were not applied to the literature search. Therefore, studies from all countries and studies in any language were eligible for this review. Furthermore, these factors did not limit the scope of this review. Third, the quality of evidence from all included studies was evaluated by using the GRADE approach [38]. This increased the transparency of the quality of included studies. Fourth, although we searched for publications from the last 10 years, our earliest study was published in 2015 [39]. Therefore, it is likely that earlier studies were not missed.

The potential methodological limitations of this systematic review should also be discussed. First, this review used a single database (ie, PubMed) to conduct the literature search. However, PubMed is the most comprehensive medical database. Most studies in other databases are also likely to be found in PubMed. Therefore, it is likely that we did not miss any studies that were relevant to our review. However, the possibility of missing a study cannot be excluded. Second, even though our search criteria allowed for the inclusion of studies from all countries, all included studies were conducted in high-income countries. Telemedicine use in high-income and low-income countries may be different, and the results of this review should be viewed as results from high-income countries. Third, this review included studies with different follow-up periods and patient populations (ie, various health conditions and age groups). Therefore, there may have been several inconsistencies between the results of each study. Furthermore, these limitations did not allow us to perform a meta-analysis [74]. Fourth, to identify the strongest available evidence, we only included randomized controlled trials that were published in peer-reviewed journals. Therefore, publication bias (ie, the tendency to report positive study results) may be present in the included studies [75].

### Conclusion

In recent years, telemedicine use among the pediatric population has become more common. Although a clear consensus on the benefits of telemedicine approaches in pediatrics has not been reached, recent literature has shown that telemedicine services are comparable to or better than in-person services. Patients and caregivers have also consistently reported that they are more satisfied with telemedicine visits than with in-person visits. This shows promise for telemedicine in pediatric settings, especially during times when social distancing is a requirement, such as the COVID-19 pandemic. Future studies should focus on improving telemedicine delivery services, people’s access to health care, the quality of telemedicine approaches, and the integration of telemedicine into in-person physician visits. Furthermore, future studies that emphasize the cost-effectiveness of telemedicine, the use of telemedicine services in rural settings, and barriers to telemedicine technology implementation are needed to analyze the true potential of telemedicine approaches for improving children’s and adolescents’ health outcomes.

## Appendix

Multimedia Appendix 1 PRISMA (Preferred Reporting Items for Systematic Reviews and Meta-Analyses) checklist.

Multimedia Appendix 2 Detailed descriptions of study outcomes.

## Abbreviations

ADHD: attention deficit hyperactivity disorder
GRADE: Grading of Recommendations, Assessment, Development and Evaluation
HbA_1c_: hemoglobin A_1c_
PRISMA: Preferred Reporting Items for Systematic Reviews and Meta-Analyses
SFD: symptom-free day
