# Supplementary material for: Telemedicine in Pediatrics: Systematic Review of Randomized Controlled Trials
Source: JMIR Pediatr Parent. 2021 Feb 24;4(1):e22696. doi: 10.2196/22696 (PMC8078694; doi:10.2196/22696)
Supplement: Multimedia Appendix 2 [file pediatrics_v4i1e22696_app2.docx]

**Supplementary File 2.** Summary of main study findings and outcomes for all included studies

| Source (Condition) | Main Study Findings |
| --- | --- |
| Cocker et al, 2019 ^39^ (Mental Health) | - The initial screening visit was completed by a greater proportion of patients in the telemedicine group than the control group. (80.49% vs 64.04%) - Patients in the telemedicine referral group took longer to complete the initial screening visit than patients in the control group. (23.6 days vs 17.1 days) - No significant difference was observed in the proportion of patients who completed the recommended intake visit after the screening visit. (80.2% vs 83.5%) - Children in the telemedicine group were more likely to complete the screening visit in the adjusted analysis as well. (odds ratio 3.17) - No significant difference was observed in the time from referral to screening visit in the adjusted analysis. - No significant difference in proportion of parents who received family centered care in both groups. *(P=*.08) - Parent’s in the telemedicine group reported higher satisfaction with the referral system and care received. - No significant difference was observed in quality of life at 6 months in both groups. |
| Erkkola-Anttinen et al, 2019 ^40^ (Otitis Media) | - Video or image was obtained in 98% of all parent-performed examinations. (median video length 18s) - 67% of all videos were of sufficient diagnostic quality.   - Diagnosis of healthy ear, acute otitis media (AOM)^[[1]](#footnote-1)^, or otitis media with effusion (OME)^[[2]](#footnote-2)^ could be made in 56% of diagnostic quality videos.   - Diagnosis could only be made in 8% of non-diagnostic quality videos.   - Diagnosis could be made in 40% of all videos received. (Most common causes: tympanic membrane (TM)^[[3]](#footnote-3)^ not visualized, improper clarity, presence of cerumen, lack of testing of TM movement) - Diagnosis of AOM could be confirmed or excluded in 87% of all videos captured during respiratory infection.   - 99% in sufficient diagnostic quality videos   - 58% in insufficient diagnostic quality videos - During week one of the intervention the immediate teaching group was taught how to perform otoscopy and delayed teaching group was not. 62% of videos in the immediate teaching group were of sufficient diagnostic quality compared to 22% in the delayed teaching group. *(P<*.001)   - Cerumen was present more frequently in the delayed teaching group videos. (47% vs 18%)   - Diagnosis was more frequently made from immediate teaching group videos. (33% vs 12%, *P*<.001)   - Detection or exclusion of AOM could be made more frequently in immediate teaching group regardless of diagnostic quality. (78% vs 30%, *P*<.001) - One week after the delayed teaching group received their education session, 64% of their videos were of sufficient diagnostic quality.   - Diagnosis could be made in 34% of their videos.   - Detection or exclusion of AOM could be made in 80% of their videos. - 78% of families reported that their child was able to learn how to calm down during the smartphone otoscopy procedure - 22% of families were able to conduct the otoscopy with only one parent. - 24% of families noted that smartphone otoscopy to be a burden on the family. - 83% of families would consider using smartphone otoscopy on a daily basis. |
| Perry et al, 2018 ^38^ (Asthma) | - No significant difference was observed in symptom free days (SFD)^[[4]](#footnote-4)^ between the intervention and usual care (UC)^[[5]](#footnote-5)^ groups post-treatment. *(P=*.51) - Patients in both groups still had uncontrolled asthma at the end of treatment. - The family activity domain of the Child Health Survey for Asthma (CHSA)^[[6]](#footnote-6)^ increased for UC group compared to intervention. *(P=*.02) - All other domains in CHSA showed no significant difference between groups. - Greater percentage of patients in the intervention group used a peak flow meter compared to UC group. *(P<*.0001) - Greater percentage of patients in the intervention group were compliant with asthma medication post-treatment compared to UC group. *(P=*.03) - No significant difference between groups on rates of controller asthma prescriptions. - No significant difference in the asthma medication ratio between groups. - No significant difference in quality of life scores from baseline for both treatment groups. |
| Halterman et al, 2018 ^37^ (Asthma) | - Children in the telemedicine group had more symptom free days (SFD) post-intervention than children in the control group. (11.6 vs 0.97, *P*=.01) - Intervention group had fewer symptom days, symptom nights, and limited activity days. - Greater proportion of patients in the telemedicine group were prescribed preventive medication. (91% vs 67%) - Lesser percentage of patients in the telemedicine group reported emergency room visits or hospitalizations. (7% vs 15%) - In the final follow-up longitudinal visit, telemedicine group had 0.85 more SFD than control and a significant treatment by time interaction. *(P<*.02) - Exhaled nitric oxide test (FeNO)^[[7]](#footnote-7)^ level decreased more in the telemedicine group than the control group. (mean difference -5.54) - Quality of life for caregivers improved in both groups, with no significant difference. - 95.7% reported that the program was helpful and 96.5% reported they would partake in another similar program. - Parents in the telemedicine group reported that the intervention helped them learn more about asthma medications (improved their communication with the school nurse (54.4% vs 40.2%) and made them more comfortable with the school nurse giving their child medication (96.9% vs 88.0%). |
| O’Connor et al, 2017 ^34^ (Skin Condition) | - Median photograph quality rating score (PQRS)^[[8]](#footnote-8)^ was 9 out of 10. - Concordance of photograph diagnosis vs in person diagnosis for all photographs was 83%.   - Concordance for photographs deemed high quality to make a diagnosis was 89%. - Mean PQRS score for photographs with a diagnosis was 8.9, whereas the mean PQRS score for photographs with no diagnosis was 7.0. - Concordance of diagnosis varied based on diagnostic category. - The group which received photography instructions had a higher average image quality score and higher mean number of images provided compared to no instructions, but this was not statistically significant. - No significant difference was observed in concordance of diagnosis for both photograph instruction and no instruction groups. *(P=*.68) - On scale of 1 (not willing) to 10 (very willing) to describe the willingness of parents to use teledermatology, the median response was an 8. - The median price parents were willing to pay for a virtual dermatology visit on a scale of 0$ to 200$ was 20$. |
| Di Bartolo et al, 2017 ^33^ (Type I Diabetes) | - HbA1C levels were reduced in both telemedicine and control groups post-treatment, with no difference between groups. *(P=*.051) - No significant difference in compliance of self-monitoring blood glucose (SMBG)^[[9]](#footnote-9)^ post-treatment between groups. *(P=*.85) - For those patients who were compliant with SMBG at 6 months, HbA1C levels decreased. - For those patients who were not compliant with SMBG at 6 months, HbA1C levels only showed minor changes. - Patients in the telemedicine group showed a greater decrease in HbA1C levels at 6 months than the control group. *(P=*.25) - The control group also switched to the experimental telemedicine meter at 6 months. At 12 months, the control group showed a decrease in HbA1C levels. *(P=*.24) - At 12 months, HbA1C levels stayed stable compared to the 6-month level for the experimental group. - Greater than 50% of patients in both groups were compliant with SMBG at 6 months. - No significant difference in SMBG compliance between groups at 12 months. *(P=*.70) - No significant difference in quality of life measures between groups at both 6 months and 12 months. - Contact rates (total of telephone calls, SMS messages, emails) were significantly higher in telemedicine group than control group. - No significant difference in incidences of hypoglycemia between groups. |
| Fleischman et al, 2016 ^32^ (Obesity) | - Group 1 (PCP visits and specialist tele-visits) showed a greater decrease in BMI z-score at 3 months than Group 2 (PCP visits only). *(P=*.049) - Group 1 had a significant decrease in BMI at 6 months, while Group 2 did not. *(P=*.0006 vs *P*=.08) No significant difference was observed between groups. - At six months, Group 1 received PCP visits only and Group 2 received PCP and specialist tele visits. - Group 1 BMI was significantly different from baseline at 9 months *(P=*.004) and 12 months *(P=*.03). - Group 2 BMI was lower and was significantly different from baseline at 12 months *(P=*.03). - No significant difference in BMI, waist circumference, and triceps skinfold between groups was observed at 12 months. - Physical activity and blood pressure did not change during the study for both groups. - Dietary glycemic load was significantly different for each group during the specialist tele-visit phase of the study. - At 6 months, patients in Group 1 found the program more helpful than patients in Group 2. *(P=*.06) - Group 1 patients were more likely to recommend to the program to others at 6 months than at 12 months. *(P=*.03) - If given the opportunity to choose between obesity specialist tele-visits or in person visits, 14 patients would choose tele-visit and 7 indicated no preference. |
| Rhodes et al, 2017 ^35^ (Obesity) | - No significant difference between or within groups for change in dietary fat content before and after treatment. - There was a lower glycemic load in the low glycemic load group compared to the low fat group after treatment. *(P=*.003) - No significant difference in glycemic load change after treatment in either group. *(P=*.06) - Low glycemic load group had a decrease in total energy intake within group post treatment. *(P<*.005) - Low glycemic load group had lower total energy intake post-treatment compared to the low fat group. *(P=*.001) - No significant difference in change in total energy intake from baseline to post-treatment between groups. *(P=*.06) - In the adjusted analysis, there was a significantly higher reduction in dietary glycemic load in the low glycemic load group than in the low fat group. *(P=*.03) - In the adjusted analysis, no significant difference was observed for reduction in dietary fat intake between groups post-treatment. - Low fat group reported higher satisfaction compared to the low glycemic load group. *(P=*.01) |
| Vander Stoep et al, 2017 ^36^ (ADHD) | - Caregivers in both the children’s ADHD telemental health treatment study (CATTS) and augmented primary care (APC) groups showed improvement in caregiver distress by the end of the study. - Caregivers in the CATTS group had significant reduction at 25 weeks in questionnaire PSI *(P<*.01), PHQ-9 *(P<*.05), and CSQ *(P<*.001) scores. (Cohen’s effect size scores 0.59, 0.27, and 0.45, respectively) - Caregivers in the CATTS group also had significant increase in questionnaire FES score at 25 weeks. *(P<*.01, Cohen’s effect size -0.44) - A significant proportion of caregiver distress levels was affected by reduction in their child’s symptoms and increase in parent role performance.   - Parenting stress (41%)   - Caregiver depression (48%)   - Caregiver strain (43%)   - Family empowerment (26%) - Only child’s ODD symptom reduction made a significant difference to caregiver distress levels. |
| Davis et al, 2016 ^31^ (Obesity) | - No significant difference in satisfaction scores between the telemedicine and telephone groups. - No significant difference in attendance between telemedicine and telephone groups. - No significant patient BMI difference pre-treatment to post-treatment between telemedicine and telephone groups. *(P>*.05) - No significant parent BMI difference pre-treatment to post-treatment between telemedicine and telephone groups. *(P>*.05) - No significant difference in group by time change, feeding assessment scale, or quality of life measures from pre-treatment to post-treatment. |
| Powers et al, 2015 ^30^ (Cystic Fibrosis, Pancreatic Insufficiency) | - After treatment, the control group had significant lower energy intake than the behavioral and nutritional treatment (BEH)^[[10]](#footnote-10)^ group. *(P<*.001) - No significant difference between groups in weight z score after treatment. *(P=*.25) - The control group had a greater decrease in height z score than the BEH group after treatment. *(P=*.49) - During follow-up, the BEH group had a greater average energy intake than the control group. *(P=*.02) - No significant difference between groups in weight z score at follow-up. *(P=*.61) - Both BEH and control groups were considered to be equally credible throughout the trial. - BEH group was seen to be more credible after treatment. - Both BEH and control groups were considered to be equally credible at follow-up. |

1 AOM: acute otitis media; 2 OME: otitis media with effusion; 3 TM: tympanic membrane; 4 SFD: symptom free days; 5 UC: usual care; 6 CHSA: Child Health Survey for Asthma; 7 FeNO: exhaled nitric oxide test; 8 PQRS: photograph quality rating score; 9 SMBG: self-monitoring blood glucose; ^10^ BEH: behavioral and nutritional treatment

1. [↑](#footnote-ref-1)
2. [↑](#footnote-ref-2)
3. [↑](#footnote-ref-3)
4. [↑](#footnote-ref-4)
5. [↑](#footnote-ref-5)
6. [↑](#footnote-ref-6)
7. [↑](#footnote-ref-7)
8. [↑](#footnote-ref-8)
9. [↑](#footnote-ref-9)
10. [↑](#footnote-ref-10)
